# Deletion at an 1q24 locus reveals a critical role of long noncoding RNA *DNM3OS* in skeletal development

**DOI:** 10.1186/s13578-021-00559-8

**Published:** 2021-03-02

**Authors:** Ting-ting Yu, Qiu-fan Xu, Si-Yang Li, Hui-jie Huang, Sarah Dugan, Lei Shao, Jennifer A. Roggenbuck, Xiao-tong Liu, Huai-ze Liu, Betsy A. Hirsch, Shen Yue, Chen Liu, Steven Y. Cheng

**Affiliations:** 1 Department of Medical Genetics, School of Basic Medical Sciences, Nanjing Medical University, Jiangsu 211166 Nanjing, P. R. China; 2 Department of Medical Genetics, Children’s Hospital and Clinics of Minnesota, Minneapolis, MI 55404 USA; 3 Department of Neurology, Ohio State University Medical Center, Columbus, OH 43210 USA; 4 University of Minnesota Medical Center-Fairview, Minneapolis, MI 55404 USA

**Keywords:** Skeletal abnormalities, 1q24, lncRNA, *DNM3OS*, Nerve growth factor

## Abstract

**Background:**

Skeletal development and maintenance are complex processes known to be coordinated by multiple genetic and epigenetic signaling pathways. However, the role of long non-coding RNAs (lncRNAs), a class of crucial epigenetic regulatory molecules, has been under explored in skeletal biology.

**Results:**

Here we report a young patient with short stature, hypothalamic dysfunction and mild macrocephaly, who carries a maternally inherited 690 kb deletion at Chr.1q24.2 encompassing a noncoding RNA gene, *DNM3OS*, embedded on the opposite strand in an intron of the *DYNAMIN 3* (*DNM3)* gene. We show that lncRNA *DNM3OS* sustains the proliferation of chondrocytes independent of two co-cistronic microRNAs *miR-199a* and *miR-214*. We further show that nerve growth factor (*NGF*), a known factor of chondrocyte growth, is a key target of *DNM3OS*-mediated control of chondrocyte proliferation.

**Conclusions:**

This work demonstrates that *DNM3OS* is essential for preventing premature differentiation of chondrocytes required for bone growth through endochondral ossification.

## Background

Development of the skeletal system that supports body structures and maintenance of its homeostatic state are highly complex processes orchestrated by an elaborate array of gene activities. A frequently occurring developmental skeletal abnormally is short stature, which can arise as part of systemic diseases [1–3]. Height gain as the result of bone elongation is driven by chondrogenesis occurring at the growth plate, which is a cartilaginous structure located near the ends of weight-bearing bones in children [4]. Thus, reduced chondrogenesis at growth plates is the main cause underlying short stature.

The rate of chondrogenesis at growth plates depends on several factors, including nutritional intake, systemic hormonal levels, as well as paracrine growth factors and the extracellular matrix signaling [4–7]. Consequently, genetic lesions that disrupt the regulation of in these systems will result in short stature. In fact, genetic testing of children with abnormal growth has identified multiple intracellular pathways that involved in chondrocyte differentiation in the growth plate. One of them is the *RAS* oncogene and the mitogen-activated protein kinase (*MAPK*) signaling pathway, which integrate signals from several growth factors such as growth hormone (*GH*), fibroblast growth factors (*FGFs*) and epithelial growth factor (*EGF*) [8–11]. Mutations in this pathway underpin a number of genetic syndromes that are collectively termed ‘rasopathies’; these include Noonan syndrome, LEOPARD syndrome, Costello syndrome, as well as cardio-facio-cutaneous and neurofibromatosis–Noonan syndrome, a common element of these syndromes is the postnatal growth failure to varying degrees [12].

Since the beginning of the new millennium, mounting evidence has shown that microRNAs and long non-coding RNAs play critical roles in regulating various cellular processes [13]). These non-coding RNAs regulate gene expression and cell signaling through diverse mechanisms. However, while lncRNAs that regulate epigenetic control of gene expression, development as well as govern human traits have been rapidly identified, few lncRNAs have been implicated in skeletal biology. Here, we reported a patient with short stature, hypothalamic dysfunction and mild macrocephaly, who carries a maternally inherited 690 kb deletion at Chr.1q24.2 encompassing a long noncoding RNA gene, *DNM3OS.* This lncRNA reading frame is of 7.8 kb in length embedded on the opposite strand within the 14th intron of *the DYNAMIN 3* gene (*DNM3)*, and carries two microRNAs: *miR-199a-5p* and *miR-214*. Our data show that it is capable of promoting primary chondrocyte proliferation and inhibiting chondrocyte differentiation via up-regulating nerve growth factor (*NGF*). Our data further show that the mouse counterpart of this lncRNA, *Dnm3os*, is a bona fide regulatory factor of skeleton development independent of its embedded co-cistronic *miR199a* and *miR214*.

## Results

### **Deletion of***DNM3OS***is associated with developmental delay**

**Fig. 1:**
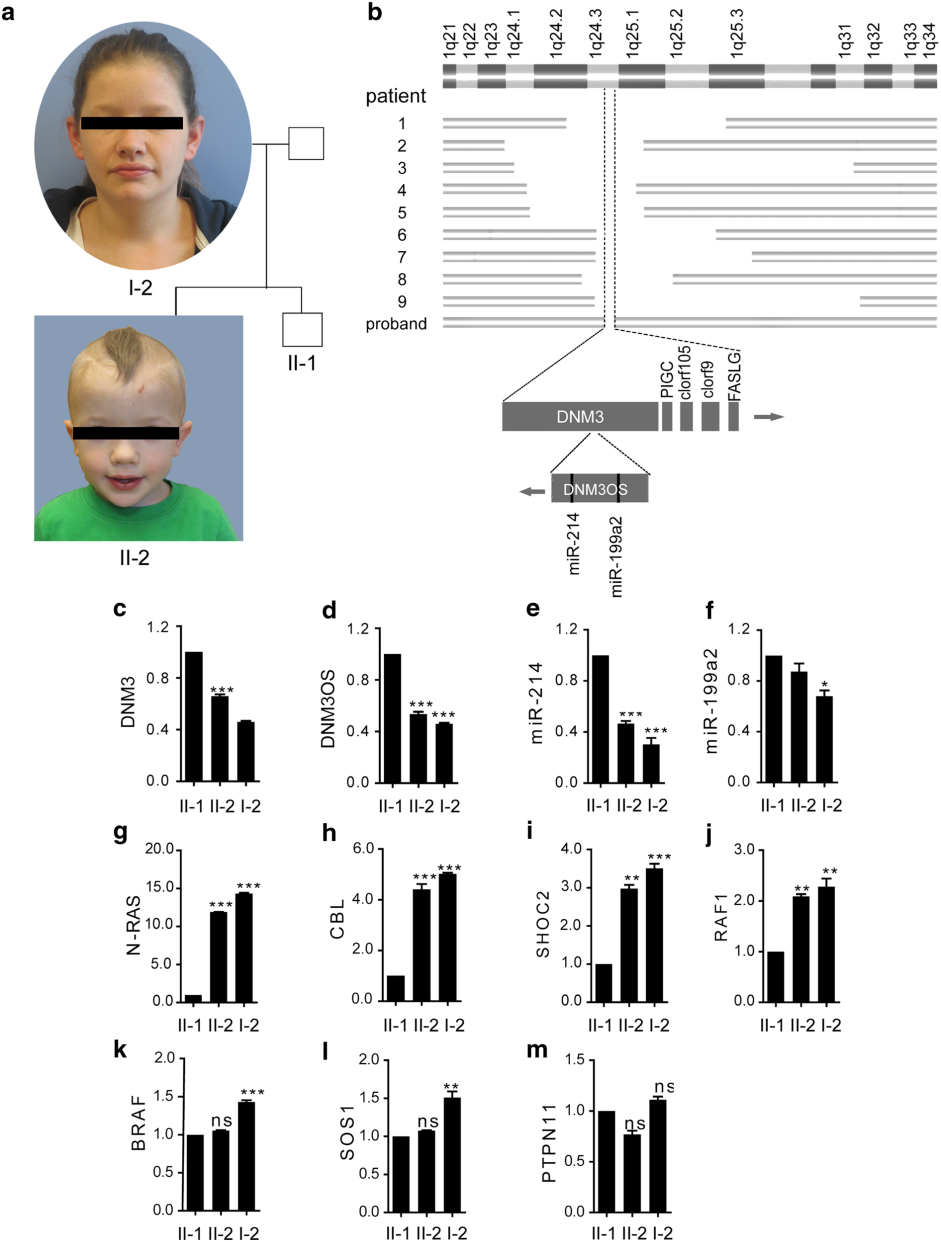
Deletion of lncRNA-*DNM3OS* is associated with autosomal dominant inheritance of 1q24 deletion syndrome phenotypes. **a** Family tree of the proband (II-2). **b** Chromosomal maps of a cohort of 9 individuals with 1q24-25 deletions. **c**–**f** RT-qPCR quantifications of *DNM3* mRNA, lncRNA-*DNM3OS*, *miR-214*, and *miR-199* in the blood. The 3’ stem-loop primer for human *miR-199* recognizes both *miR-199a* and *miR-199b* on chromosome 9, the latter of which is not affected by the deletion. **g**–**m** RT-qPCR quantifications of *N-RAS*, *CBL*, *SHOC2*, *RAF1*, *BRAF*, *SOS1*, and *PTPN11* in the blood. One-way ANOVA test was used for statistical analysis. **P < 0.01, ***P < 0.001, and ns, not significant

A young boy was presented to us with clinical manifestation of short stature, hypothalamic dysfunction and mild macrocephaly reminiscent of Noonan syndrome (Fig. 1a, and Additional file 1). The birth weight and height of the proband (II-2) were both below the 5th percentile among Caucasians, and radiographic examinations first conducted at 2 years of age indicated delayed bone growth; however, laboratory tests found no hormonal imbalance, and tests for mutations in 12 known genes compiled in the Noonan Spectrum Chip were also negative (data not shown). Using oligonucleotide-based array comparative genomic hybridization, we identified a maternally inherited 690 kb deletion at 1q24.3 (Fig. 1b), which falls within the common chromosomal deletion interval reported in a cohort of 9 patients with facial features, prenatal-onset short stature with delayed bone age, single palmar crease, and brachydactyly similar to the proband [14]. Deletions around 1q24-1q25 have been noted for growth deficiency among a myriad of symptoms [15]. Among 5 protein-coding genes and undefined open reading frames in the deleted region, we were drawn to a transcription unit called *DNM3OS* on the opposite strand in the 14th intron of *DNM3*, which encodes no protein but two microRNAs, *miR-199a-5p* and *miR-214* (Fig. 1b). In the literature, homozygous deletion of murine *Dnm3os* was reported to cause severe skeletal defects in new born mice including cranial deformity, which was most likely the cause of postnatal lethality, but it was not clear whether the long noncoding RNA *Dnm3os* as a whole, or *miR-199a-5p* or *miR-214* plays that essential role for the skeletal development [16]. Previously we demonstrated that *miR-214* downregulates *N-ras* to promote myogenic differentiation at the expense of osteogenic differentiation [17]; however, genetic ablation of *miR-214* did not lead to obvious signs of Noonan syndrome-like features including growth delay in mice [18, 19] (Additional file 2: Fig. S1). Nevertheless, expression of 7 Noonan syndrome genes in the *Ras* pathway was drastically increased (Additional file 3: Fig. S2) in MEF cells isolated from *miR-214* knock-out mice, in keeping with the presence of *miR-214* recognition sites in the 3’UTR or coding regions of these RNA transcripts (Additional file 4: Table S1). Using real-time PCR, we found that the levels of *DNM3*, *DNM3OS*, and *miR-214* RNA transcripts in the peripheral blood of the proband (II-2) and his carrier mother (I-2) were only 50% of those in his non-carrier brother (II-1) (Fig. 1c–f). Interestingly, the peripheral expression of 4 out of the same 7 Noonan syndrome genes tested in *miR-214* KO mice was significantly increased in carriers II-2 and I-2 relative to the non-carrier II-1 (Fig. 1g–m). These data suggest that *DNM3OS* carries an essential function that accounts for the roles of chromosomal 1q23-1q25 interval in skeletal development and although the ablation of *miR-214* in mice is not sufficient to cause phenotype that resembles any aspect of the partial Noonan syndrome manifestation seen in the proband, it nevertheless partakes in the regulation of skeletal growth via the *Ras* pathway.

### **Murine***Dnm3os***is required for maintaining the proliferative potential of articular chondrocytes**

**Fig. 2:**
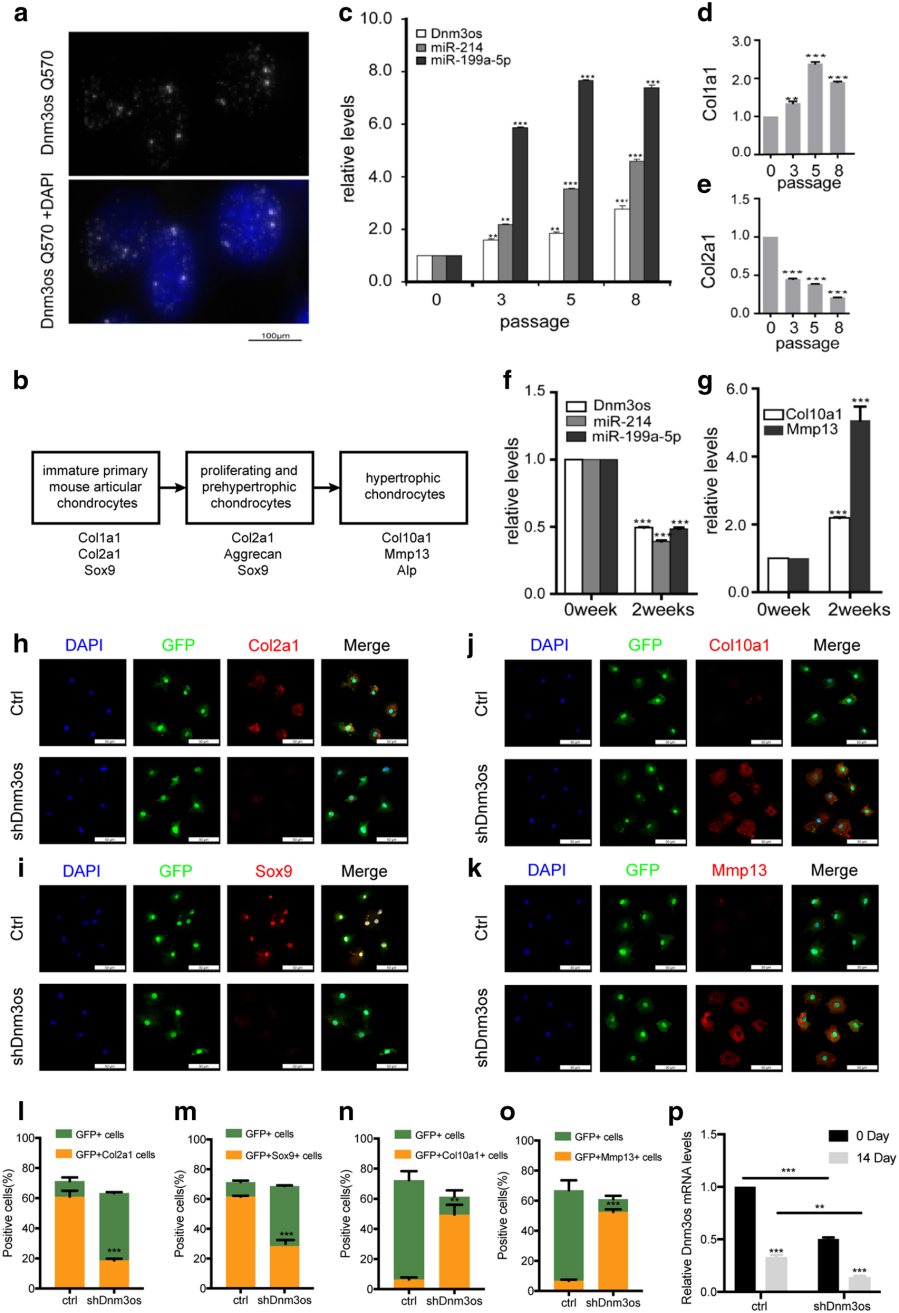
LncRNA- *Dnm3os* is required for maintaining the proliferative potential of chondrocytes. **a** RNA-FISH detection of lncRNA-*Dnm3os* in NIH3T3 cells using Quasar 570 labelled Stellaris oligonucleotide probe. **b** Flow chart of in vitro chondrogenic differentiation. **c** RT-qPCR detection of RNAs in primary articular chondrocytes at passages as noted. **d**, **e** RT-qPCR detection of proliferating chondrocyte marker, *Col2a1*, and osteoblast marker *Col1a1*, respectively; the latter cell descends from mesenchymal lineage. **f** RT-qPCR detection of RNA and **g** hypertrophic chondrocyte markers *Col10a* and *Mmp13* following differentiation for 2 weeks. **h**–**k** IF staining of *Col2a1*, *Sox9*, *Col10a1*, and *Mmp13* and **l**–**o** quantification thereof following differentiation for 2 weeks. Prior to induction of differentiation, the primary articular chondrocytes were transfected with scrambled shRNA (ctrl) or sh*Dnm3os*, both of which carried a GFP marker. **p** RT-qPCR detection of *Dnm3os* at the start and end of in vitro chondrogenic differentiation. Student T-test was used for statistical analysis. **P < 0.01, ***P < 0.001

Both human and mouse *Dnm3os* encodes a 7.8 kb RNA transcript, which was identified due to its depleted expression in mouse limb buds lacking basic helix-loop-helix transcription factor *Twist * [20]. First appearing in limb buds and other future skeletal elements [17], robust *Dnm3os* expression was reported in perichondrial cells and periarticular chondrocytes at the cartilage growth plate [16]. Using RNA-FISH, we found that full length *Dnm3os* RNA is confined to the nucleus (Fig. 2a). During embryonic development, chondrocytes in the long bone growth plate undergo an orderly proliferation and differentiation process that eventually gives rise to trabecular bones [21]. This chondrogenic process can be faithfully recapitulated in vitro using isolated immature primary mouse articular chondrocytes (iMAC) [22]. We observed that expression of *Dnm3os* and its co-cistronic *miR-199a-5p* and *miR-214* progressively increased (Fig. 2b, c) following several passages in the maintenance medium as the cells gradually reverted to a “dedifferentiated” state marked by the switch of cell surface collagen types from *Col2a1* to *Col1a1* (Fig. 2d, e). Correspondingly, the expression of chondrogenic transcription factor *SOX9* gradually decreases with passaging (Additional file 5: Fig. S3A). When introducing sh*Dnm3os*, the switch of cell surface collagen types from *Col2a1* to *Col1a1* was slightly slowed, suggesting that *Dnm3os* was not a key fact for dedifferentiated (Additional file 5: Fig. S3B). Conversely, expression levels of these non-coding RNAs all decreased dramatically after the cells were induced to differentiate into hypertrophic chondrocytes marked by *Col10a1* and *MMP13* (Fig. 2f, g). These spatial and temporal expression patterns of *Dnm3os* are consistent with an essential role in promoting the proliferation but suppressing the differentiation of chondrocytes. To ascertain such a function, we silenced *Dnm3os* expression using shRNAs in the iMAC before inducing their differentiation. At the end of two weeks, immunofluorescence microscopy indicated that over 60% of scrambled shRNA transfected cells (marked by GFP) still retained *Col2a1* and *Sox9*, but these percentages dropped to below 20 and 40%, respectively, in the cells transfected with sh*Dnm3os* (Fig. 2h, i, l, m). Remarkably, many GFP positive cells that received sh*Dnm3os* completely lost *Col2a1* and *Sox9* (Fig. 2h, i, l, m), indicating that they had differentiated further into hypertrophic chondrocytes (Fig. 2b). Elevated expression of *Col10a* and *Mmp13* confirmed the role of silencing *Dnm3os* in promoting chondrogenic differentiation (Fig. 2j, k, n, o). The expression level of *Dnm3os* maintained at a relative low level in the cells transfected with sh*Dnm3os* (Fig. 2p). Moreover, EdU assay showed *Dnm3os* silencing decreased the proliferation of iMAC by 10% (Additional file 5: Fig. S3 C–E).

**Fig. 3:**
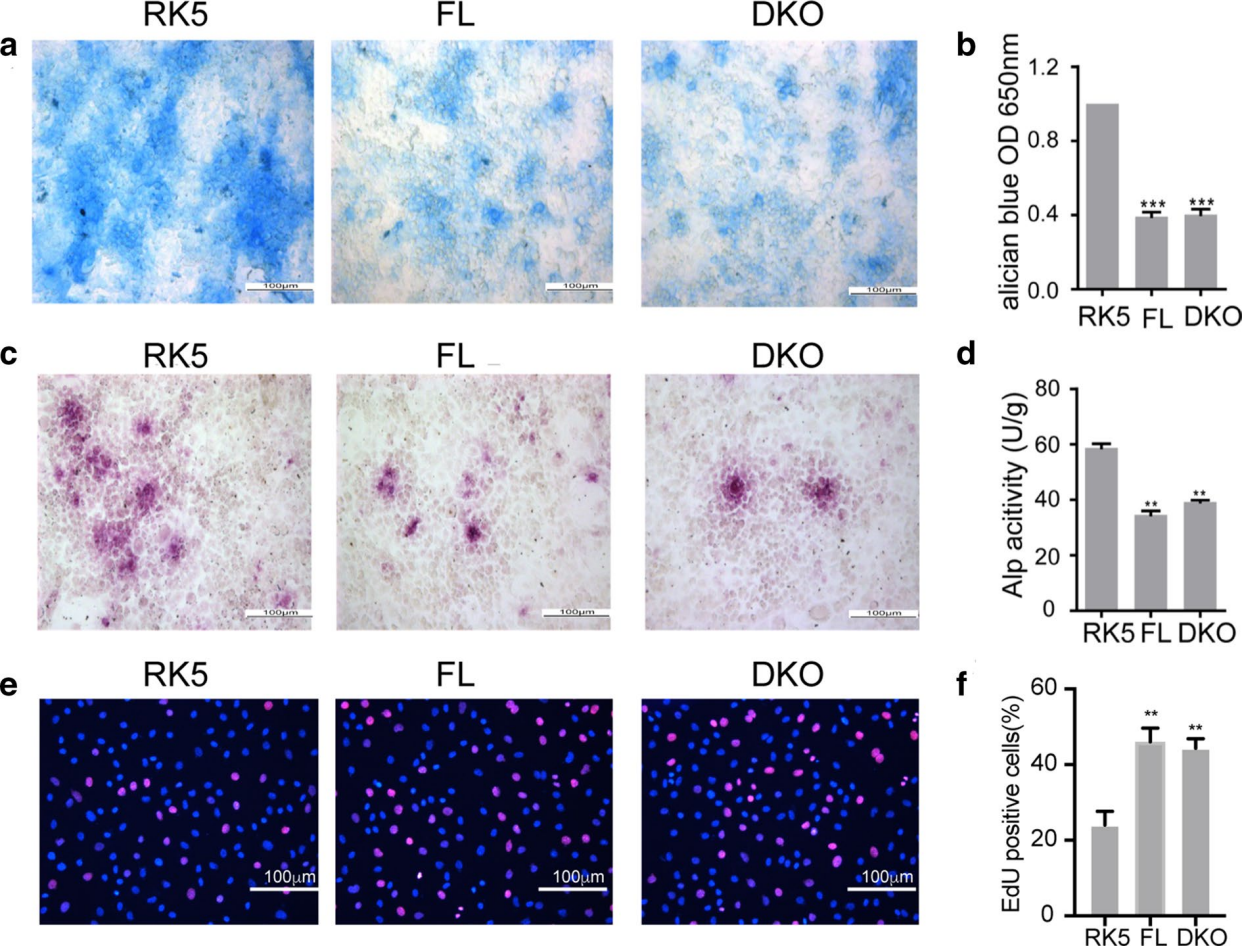
LncRNA-*Dnm3os* sustains the proliferation of chondrocytes independent of the co-cistronic microRNAs. **a** Alcian blue staining and **b** quantification thereof of cartilage matrix produced by differentiated chondrocytes. **c** Alkaline phosphatase staining and **d** quantification thereof of differentiated chondrocytes. The primary articular chondrocytes were transfected with the control vector or constructs that express FL or the mutant *Dnm3os* prior to induction of chondrogenic differentiation. **e** EdU labeling of proliferating chondrocytes and **f** calculation thereof. The primary articular chondrocytes were transfected as in (**a**–**c**), and one day later the cells were labeled with EdU for 8 hours before visualization. The frequency of nuclear EdU labeling is determined by examination of at least three random fields, magnification ×400 and at least 300 cells and nuclei in each group. One-way ANOVA test was used for statistical analysis. **P < 0.01, ***P < 0.001

In addition to controlling *Ras* pathway genes (Additional file 3: Fig. S2), forced expression of *miR-214* and *miR-199a* blocked chondrogenesis (Additional file 6: Fig. S4A–F) and promoted proliferation (Additional file 6: Fig. S4G–H). Thus, to determine if the large *Dnm3os* is sufficient in regulating chondrogenesis or requires the two co-cistronic microRNAs, we cloned the mouse full length *Dnm3os* (FL) and generated a mutant that lacks both microRNAs (DKO). RT-QPCR analysis confirmed the overexpression of FL and DKO in iMAC cells (Additional file 5: Fig. S3H). Forced expression of either FL or the DKO mutant *Dnm3os* impeded the chondrogenesis as evident by reduced staining by alcian blue (Fig. 3a, b) or of alkaline phosphatase (Fig. 3c, d), which mark the cartilage matrix proteins and the mature hypertrophic chondrocytes, respectively. The effects of DKO in chondrogenesis were not due to the dominant-negative inhibition of endogenous *Dnm3os* function, since overexpression of DKO inhibited chondrogenesis as shown by decreased *Mmp13*and *Col10a1*in iMAC cells silencing endogenous *Dnm3os* with sh*Dnm3os* transfection (Additional file 5: Fig. S3F–G). Forced expression of the DKO mutant also showed similar propensity to down-regulate the same cohort of *Ras* pathway genes as the parental RNA (Additional file 7: Fig. S5), indicating that *Dnm3os* can function independently of the two microRNAs. Finally, to ascertain if *Dnm3os* directly promotes the proliferation of articular chondrocytes, we labeled the cells with EdU and found that both the FL and DKO mutant *Dnm3os* accelerated the cell growth (Fig. 3e, f).

### *Dnm3os***is required for maintaining the proliferative potential of ATDC5 cells**

**Fig. 4:**
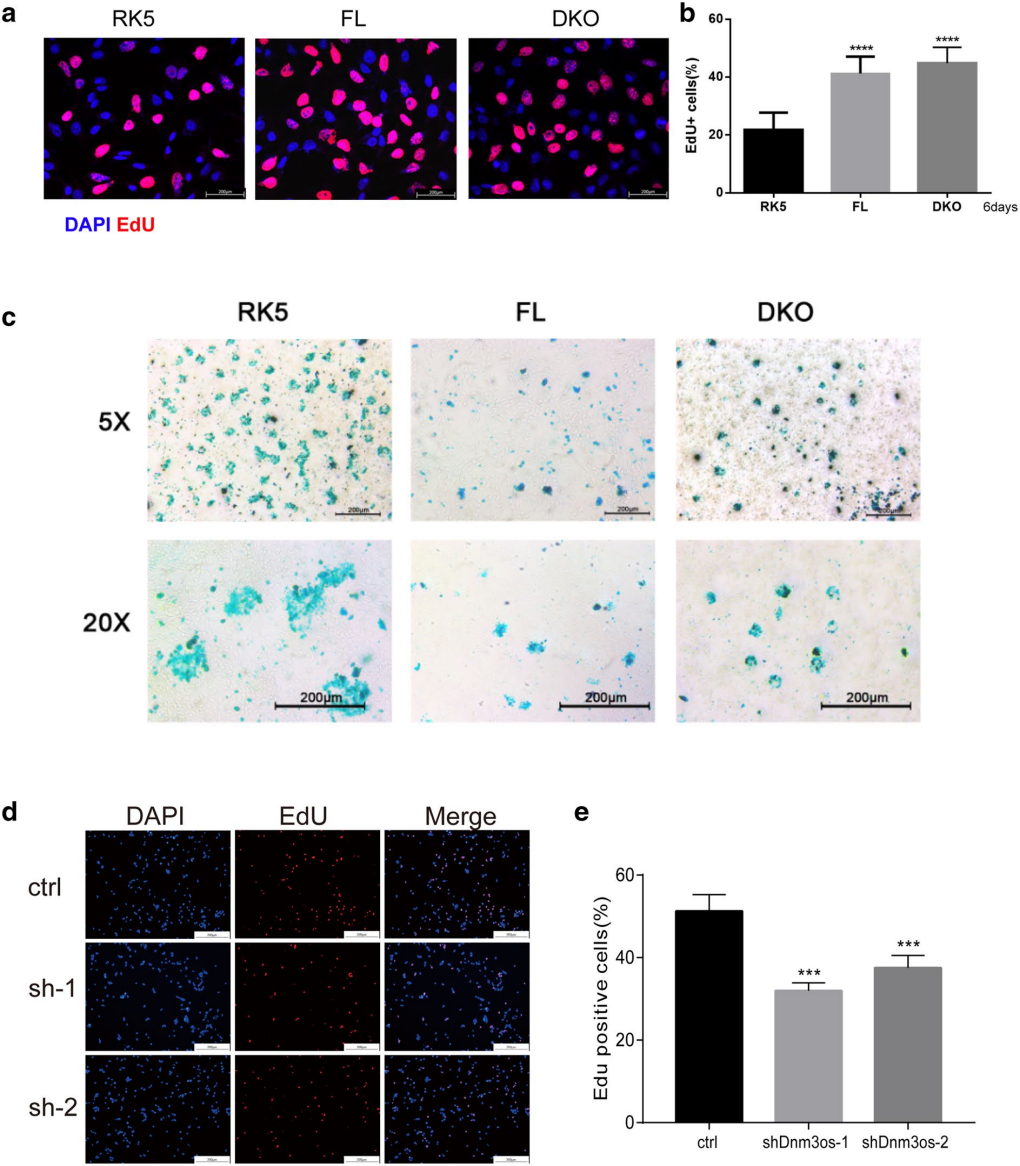
LncRNA-*Dnm3os* is required for maintaining the proliferative potential of the ATDC5 cells. **a** EdU labeling of proliferating ATDC5 cells and **b** calculation thereof. The ATDC5 cells were transfected by RK5, FL and DKO individually, and one day later the cells were labeled with EdU for 8 hours before visualization. **c** Alcian blue staining of cartilage matrix produced by differentiated ATDC5 cells. **d** EdU labeling of proliferating ATDC5 cells and **e** calculation thereof. The ATDC5 cells were transfected with scrambled shRNA, sh*Dnm3*os-1 and sh*Dnm3*os-2 individually, and one day later the cells were labeled with EdU for 8 h before visualization. The frequency of nuclear EdU labeling in (**a**) and (**d**) were determined by examination of at least three random fields, magnification ×400 and at least 300 cells and nuclei in each group. One-way ANOVA test was used for statistical analysis. ***P < 0.001, ****P < 0.0001, and ns, not significant

To corroborate the above observation in primary mouse articular chondrocytes, we took the advantage of ATDC5 cells that are widely used as an in vitro model for chondrocyte differentiation to test the pro-proliferative role of *Dnm3os*. Alcian blue staining images collected by light microscopy over an 18-day time-course indicated that insulin-supplemented differentiation medium cultivation successfully induced the differentiation of ATDC5 cells to mature hypertrophic chondrocytes (Additional file 8: Fig. S6A). Decreased *Col2a1* and increased *Col1a1* expression confirmed the differentiation on the biomarker (Additional file 8: Fig. S6B, C). Consistent with primary mouse articular chondrocytes, the expression of *Dnm3os* in ATDC5 cells gradually decreased with the chondrogenic differentiation (Additional file 8: Fig. S6D). Overexpression of FL and the DKO mutant *Dnm3os* activated ATDC5 cells proliferation and impeded the chondrogenesis as evident by 2-fold increased EDU positive cells (Fig. 4a, b and Additional file 5: Fig. S3I) and absent Alcian blue staining (Fig. 4c), while silencing *Dnm3os* with shRNA inhibited the proliferation of ATDC5 cells (Fig. 4d, e). We further employed RT-QPCR to examined the expression of *Sox9* in FL and DKO transfected ATDC5 cells and iMAC cells. Both overexpression of FL and DKO increased the expression level of *Sox9* in ATDC5 cells, while the effect in iMAC cells were barely observed (Additional file 8: Fig. S6E, F). However, the expression of *Sox9* decreased with iMAC cells passaging (Additional file 5: Fig. S3A), which suggested the overexpression of FL and DKO to some extent counteracted the reduction of *Sox9* caused by passage. These data suggest that *Dnm3os* contributes to the maintenance of ATDC5 and iMAC cells as chondrogenic progenitors and plays an independent role in regulating chondrogenesis instead of simply acting as a precursor of miRNAs.

### *NGF***is a potential***Dnm3os***-regulated gene in chondrocytes**

**Fig. 5:**
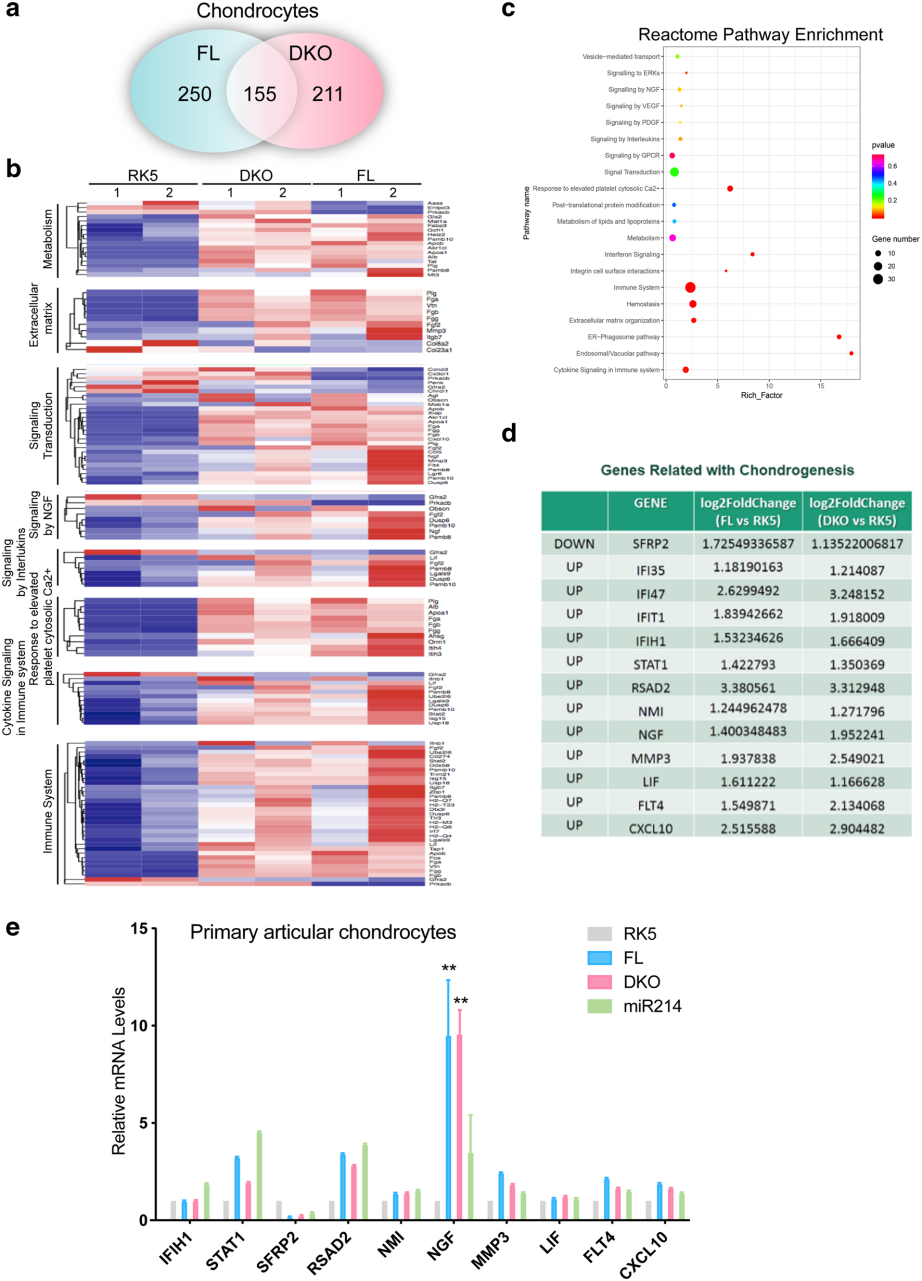
RNA-sequencing analysis of LncRNA-*Dnm3os* regulated genes in chondrogenesis. **a** Quantitative RNA-seq comparison of differentially expressed genes in FL and DKO transfected primary articular chondrocytes. Total number of genes with at least 1.15-log2Fold Change are indicated. **b** Heat map representation of the top 8 enriched pathway with highest fold change in FL and DKO transfected primary articular chondrocytes. **c** Top 20 enriched Reactome pathways in FL and DKO transfected primary articular chondrocytes. The size and color of the dots represent the enriched gene number and the range of p values, respectively. **d** Genes associated with chondrogenesis that are up- or down-regulated in FL and DKO transfected primary articular chondrocytes. **e** Verification of LncRNA-*Dnm3os* regulated genes in (**d**) by RT-qPCR in chondrocytes transfected by RK5, FL, DKO and miR214 individually. Student T-test was used for statistical analysis. *P < 0.1 and **P < 0.01

To substantiate the direct link between *Dnm3os* and chondrogenesis, we performed RNA-seq in FL and DKO mutant *Dnm3os* transfected primary mouse articular chondrocytes with 2 biological replicates. The result showed 250 and 211 differentially expressed genes in FL and DKO overexpressed cells relative to the RK5 transfected control cells, respectively (Fig. 5a), and of these, 155 were commonly expressed in those two cells transfected with *Dnm3os* vectors, suggesting that this lncRNA functions as an independent regulator of chondrocytes (Fig. 5a) and DKO acts as a regulator at the transcriptional level. To predict the biological function of *Dnm3os*, we categorized these 155 genes into defined pathways and we found cell cycle appeared in the top altered pathways, which confirmed the proliferation effect of *Dnm3os* (Additional file 8: Fig. S6G). To further provide biological insight to the differential expression genes, the 155 genes were assigned into defined pathways by using Reactome databases (Fig. 5b). Among the top 20 significantly altered pathways ranked in the dot plot enrichment map, signal transduction, metabolism and extracellular matrix organization, whose dynamics are key to tissue morphogenesis appeared with majority gene enrichment and statistical significance. We further dissected 8 of the top enriched pathways and found matrix metalloproteinase 3, collagen genes including *Col8a2*, *Col23a* and growth factors including *NGF*, *FGF*, as visualized by the heat map (Fig. 5c). The expression changes of these genes suggested the ongoing extracellular matrix remodeling and accelerated cell proliferation. Subsequently, we picked up 14 genes which have been reported to play a role in chondrocyte proliferation or differentiation and verified the change by QPCR analysis (Fig. 5d, e). To exclude the *miR*-*214* target genes, we detected the expression of these 14 genes in *miR-214* transfected ATDC5 cells simultaneously, and found *NGF* was a potential target gene regulated by lncRNA-*Dnm3os* specifically (Fig. 5e).

### *Dnm3os***-induced***NGF***maintains the proliferative potential of ATDC5 cells**

**Fig. 6:**
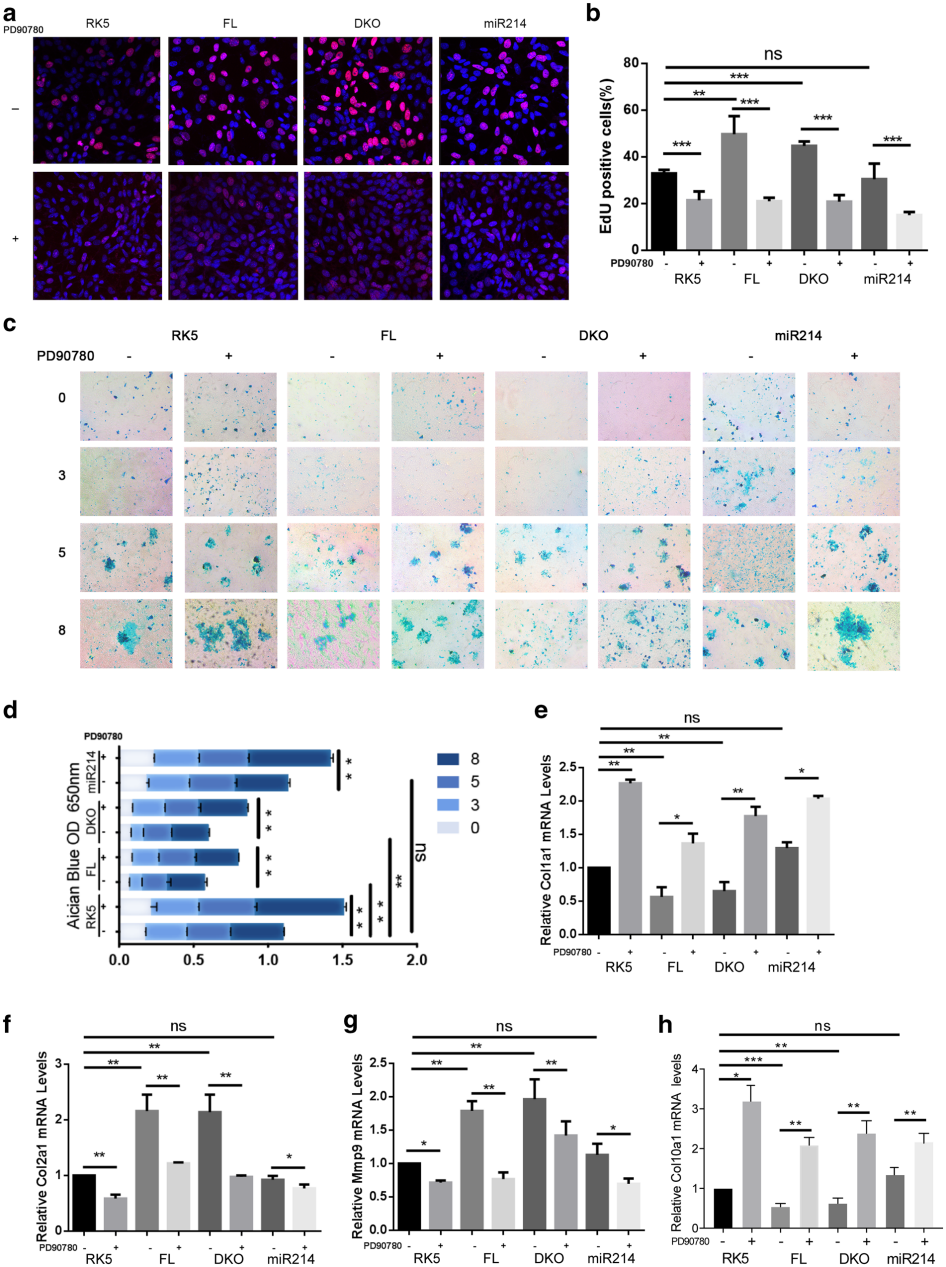
LncRNA- *Dnm3os* sustains the proliferation of chondrocytes by upregulating *NGF*. **a** EdU labeling of proliferating Atdc5 cells and **b** calculation thereof. The ATDC5 cells transfected by RK5, FL, DKO, and miR214 were treated with vehicle of PD90780 (1‰ DMSO) or PD90780 (10ug/1ml) for 24 hours, and one day later the cells were labeled with EdU for 8 h before visualization. The frequency of nuclear EdU labeling is determined by examination of at least three random fields, magnification ×400 and at least 300 cells and nuclei in each group. **c** Alcian blue staining and **d** quantification thereof of cartilage matrix produced by the differentiated ATDC5 cells. The ATDC5 cells were transfected and treated with PD90780 as in (**a**). **e**–**h** RT-qPCR quantifications of *Col1a1*, *Col2a1*, *Mmp9* and *Col10a1* in ATDC5 cells transfected and treated with PD90780 as in (A). Student T-test was used for statistical analysis. *P < 0.1, **P < 0.01, ***P < 0.001, and ns, not significant

As a neuropeptide, *NGF* was involved in cartilage metabolism and is reported to mediate the chondrogenic differentiation of mesenchymal stem cells [23]. To determine if *NGF* play a role in *Dnm3os* mediated chondrocytes proliferation, we treat FL, DKO mutant and *miR*-*214* transfected ATDC5 cells with PD90780, an inhibitor of *NGF*. 24 hours after treatment, we labeled the cells with EdU and found that inhibition of *NGF* blocked FL and DKO mutant *Dnm3os*-induced chondrocytes proliferation (Fig. 6a, b). On the other hand, the chondrogenesis was promoted by PD90780 as evident by increased staining by alcian blue in FL and DKO mutant *Dnm3os* transfected cells after 18-days differentiation (Fig. 6c, d). Consistently, FL and DKO mutant *Dnm3os*-induced collagen types switch from *Col1a1* to *Col2a1* was interrupted by PD90780 (Fig. 6e, f). *MMP9*, a target gene of *NGF* was downregulated with treatment of PD90780 and validated the inhibitory effect of the compound (Fig. 6g). RT-QPCR analysis of *Col10a1* confirmed the that inhibition of *NGF* in FL and DKO constructs overexpressing cells promoted differentiation (Fig. 6h). These data collectively indicated these chondrocytes have been released from the bondage of *Dnm3os* and entered the differentiation state.

## Discussion

Skeletal development is a complex process that exquisitely controlled both spatially and temporally by cell signaling networks and gene regulation programs. In the past decades, studies of congenital human disease reveal a great deal of genes that involved in bone growth including *Ras-MAPK* pathway, *Wnt* and *Hedgehog* signaling [24, 25]. However, the contribution of non-coding RNA especially lncRNA to skeletal development still remains unclear. Here, we present evidence that shows LncRNA-*Dnm3os* is required for maintaining the proliferative potential of articular chondrocytes, and we demonstrate that *Dnm3os* has specific gene targets such as *NGF*, which impeded chondrocytes to differentiation state. Thus, *Dnm3os* defines a new class of lncRNAs that serve as transcriptional regulator in addition to produce microRNAs, thereby forming a regulatory network that maintains a proper pool of proliferating chondrocytes to supply bone growth through endochondral ossification, which account for the short stature.

In 2011, Burkardt et al. reported nine patients with a core clinical symptom of mental retardation, microcephaly accompanied by short stature, and identified a crucial deletion region spanning 1.9 Mb at 1q24.3q25.1 [14]. The deleted region contains 13 genes including *Dynamin 3* (*DNM3*) and *CENPL*,which encodes a protein essential for centromeric function, mitotic progression and synaptic reaction. Later, Ashraf T described 2 patients with 1q24 microdeletions and the skeletal phenotype, but had normal intellect or mild learning impairment [26]. Genetic testing of these 2 patients narrows the skeletal abnormalities to a region containing only *DNM3* and a transcript union in the opposite strand of *DNM3*, which called *DNM3* opposite strand (*Dnm3os*). This unit can be transcribed into a long non-coding RNA *Dnm3os* (LncRNA-*Dnm3os*), which was described as a precursor of two microRNAs: *miR*-*199a*-*5p* and *miR*-*214*. It was reported that *Dnm3os*, *miR214* and *miR199a*-*5p* are abundant in the skeleton system containing limb and skull [27]. *Dnm3os* deletion mice exhibited several skeletal abnormalities, including craniofacial hypoplasia and defects of dorsal neural arches [16]. Down regulation of *miR199a*-*214* cluster, especially *miR*-*214* was considered to be responsible of the phenotype. While for quite a long time, LncRNA-*Dnm3os* was described as a precursor of these two miRNAs. However, *miR*-*214* KO mice were born at Mendelian ratios and displayed a minimal reduction in body weight compared with WT littermates. Our experiments revealed scientific explanations for these paradoxical phenomena. First, LncRNA-*Dnm3os* may have compensatory effect of skeletal development since LncRNA-*Dnm3os* shares most of the target genes with *miR-214*, and genetic deletion of the *miR199a*-*214* cluster did not abolish the regulatory effect of LncRNA-*Dnm3os* (Figs. 3 and 4). The other possible explanation is LncRNA-*Dnm3os* has its own target genes which involved in skeletal development. Thus, the *Dnm3os* transcript unit defines a regulatory network between lncRNA and miRNAs.

During the past decades, emerging evidence suggests that lncRNAs can play a crucial role in manipulating various cellular processes. In particular, lncRNAs can serve as mater gene regulators at transcriptional and posttranscriptional levels,participating in embryonic development and occurrence of diseases. In some cases, lncRNAs can act as baits of microRNA and sequestrate microRNA for target mRNAs transcriptional repression. Other lncRNAs including *BACE1 AS* regulate gene expression by competing with miRNAs. And for some lncRNAs, their degradation can be triggered by microRNA. Recently, research works defined the interaction between nucleolin, *ILF*-2 and lncRNA-*Dnm3os* by RNA pull-down assays with macrophage nuclear lysates, which indicates that lncRNA-*Dnm3os* is more than a precursor of microRNAs. Due to the obvious and direct link to vertebrate skeleton development, lncRNA-*Dnm3os* may also have unique function in this biological process.

## Conclusions

This study demonstrated that lncRNA-*DNM3OS* maintains the proliferation and restrains premature differentiation of chondrocytes independent of the co-cistronic microRNAs *miR*-*199a* and *miR*-*214*. In addition, mechanistic studies showed *NGF* as a key target of lncRNA-*DNM3OS* that supports chondrocyte proliferation. Combined with our findings, lncRNA-*DNM3OS* likely plays an important role in regulating skeletal development by triggering *NGF* signaling. Future studies are required to ascertain whether there are more particular genes or signaling pathways regulated by lncRNA-*DNM3OS*.

## Materials and methods

### Single family case studies

The studies of this case obtained the informed consent from all subjects. For publication of photos, consent was obtained from the patients.

### Animals

The studies with animals follow the guidelines and ethical regulations. The research program and study protocols were approved by Animal Care and Ethical Committee of Nanjing Medical University (approval number 14,030,111).

## Isolation of mouse articular chondrocytes and culture

Primary chondrocytes were isolated from 5 to 6 days old mice as described. Briefly, cartilages from tibial plateaus and femoral condyles were excised and all extraneous soft tissues were removed. To isolate chondrocytes, the cleaned cartilages were digested two consecutive times in cell culture medium with 3 mg/ml collagenase D (Roche, Indianapolis, 11088858001) at 37 °C for 45 min, and then overnight with 0.5 mg/ml collagenase D. The next day, the dislodged cells were passed through a 2 ml Pasteur pipet successively to disperse aggregates, then through a sterile 48-µm mesh before collected by centrifugation for 10 min at 400×*g*, 20 °C. The cells were plated out at a density of 25 × 10^3^/cm^2^. Chondrogenic differentiation was induced by replacing culture medium with DMEM/F12 (1:1) (Gibco,C11330500BT), supplemented with 10% FBS, 1% pen&strep (Gibco, 15140122), 10 µg/ml insulin, 10 µg/ml transferrin, and 3 × 10 ^− 8^ M sodium selenite (Sigma, I3146). The differentiation medium was changed every 3 days.

### Plasmids construction and cell transfection

The DNA fragment containing full length *Dnm3os* sequence was amplified by PCR from mouse genomic DNA and inserted between the BamHI and SalI sites in the pRK5 vector to generate pRK5-*Dnm3os*. PCR-based deletion strategy was used to generate the *miR-199a-5p* and *miR-214* deletion mutant (DKO). The PCR primers used are as follows.

FL-F:TTCCTGGTCCTAAATTCATTGCCAG

FL-R:ATAGGAATAAAAATTACAAGTATGAA

MiR-199a2D-F1: TTCCTGGTCCTAAATTCATTGCCAG

MiR-199a2-R1:ACAGGATTTTCCACACACCGA

MiR-199a2D-F2:ACGCCATGGACGGCTGGGGACACA

MiR-199a2D-R2: ATAGGAATAAAAATTACAAGTATGAA

MiR-214D-R1: AACCTGAAGGACCCAAG

MiR-214D-F1:AAAACCTACCCGAAGTAAAG

Sh*Dnm3os* was designed by using online tools available from . Oligonucleotides (listed in Additional file 9: Table S2) of sh*Dnm3os* were cloned into pRetro-H1G shRNA expression vector. ShRNA with the sense sequence 5′-agatctTTAGTATAGATAATATTTCtacctgacccataGAAATATTAT CTATACTATTTTTggtacc-3′ which lacks complementary sequences in the human genome, was used as control (scrambled shRNA).

Primary articular chondrocytes were transfected with endotoxin-free plasmid constructs using Lipofectamine (Invitrogen,11514-015) according to the manufacturer’s procedure.

## **Blood RNA extraction and RT-qPCR**

10 ml blood from each subject was drawn with a BD Vacutainer CPT Cell Preparation Tube containing sodium citrate. Lymphocytes and monocytes were separated from the plasma in Ficoll solution (Sigma, F2637). Briefly, the blood samples were diluted with 1:1 sterile PBS, and then carefully poured onto 10 ml Ficoll solution in a 50 ml centrifuge tube (the blood must remain on top, do not mix). The tubes were centrifuged for 20 min at 350xg, and lymphocytes and monocytes between plasma and Ficoll layers were harvested using a sterile pipette. The cells were washed twice with PBS, and the RNA was extracted using the RNAiso reagent (Takara,9109) to template cDNA synthesis using the PrimeScript RT reagent kit (Takara,RR014). SYBR green real-time qPCR reactions (Vazyme,Q111) were carried out on a ABI7500 Real-Time PCR system. The cycling condition was 95°C for 5 minutes, followed by 40 amplification cycles of 95°C, 15 second and 60°C, 1 minute. For each data point, triplicate reactions were carried out and the experiment was repeated three times to assess the statistical significance. RT-qPCR primer sequences are listed as follows.

PCR primers for human genes

h*NRAS*-F: AACAAGCCCACGAACT

h*NRAS*-R: TGGCAATCCCATACAA

h*CBL*-F: CCACTTGCTCGTCTCC

h*CBL*-R: AACAGTAGTATCCCACATC

h*BRAF*-F: CCTCATTACCTGGCTCAC

h*BRAF*-R: TCTCCCAATCATCACTCG

h*RAF1*-F: GTCACGCTGGAGTGGTTCT

h*RAF*-R: ACAATACGATGCCATAGGAGT

h*SHOC2*-F: TTTTGTCCAGGCTTGAGT

h*SHOC2*-R: CATCTTTGGCATCTTTCC

h*SOS1*-F: CTTAGGTGGAGGTGAGAA

h*SOS1*-R: TGGTCCCTGATTAAATAGA

h*PTPN11*-F: TATCCTCTGAACTGTGCAGATCC

h*PTPN11*-R: TCTGGCTCTCTCGTACAAGAAAA

h*DNM3*-F: AATCCGTCCACTAGAATCCTCA

h*DNM3*-R: GGTCCATACATGCGACTACTCA

h*DNM3OS*-F: GGTCTCACCCTGCTTGTTAATCAA

h*DNM3OS*-R: TCCTGTTGTTACTGGCCCTCATGC

PCR primers for mouse genes

m*Nras*-F: CCTTGACCCGTTTGACACT

m*Nras*-R: AACCACCTACATACCTACAT

m*Cbl*-F: AGGGTTTCACCGTCTT

m*Cbl*-R: CTGGGCTGAGTGTAGTTT

m*Braf*-F: ACCTCGTCACAGTTCTCCT

m*Braf*-R: TTCTTGGCTTGAAGTTGC

m*Raf*-F: TGCGTCGGATGCGAGAAT

m*Raf1*-R: TGAGGAAGGGCTGGAGG

m*Shoc2*-F: TCGCTTTAATCGCATAAC

m*Shoc2*-R: TGAGCTACATCCAGGGTA

m*Ptpn11*-F: GAGGAGTCGATGGCAGTT

m*Ptpn11*-R: CTGAATCTTGATGTGGGTAA

m*Mmp13*-F: GTTGACAGGCTCCGAGAAAT

m*Mmp13*-R: CATCAGGCACTCCACATCTT

m*Col10a1*-F: AAGGAGTGCCTGGACACAAT

m*Col10a1*-R: ATGCCTGGGATCTTACAGGT

m*Sox9*-F: CGGAACAGACTCACATCTCTCC

m*Sox9*-R: GCTTGCACGTCGGTTTTGG

m*Col1a1*-F: GCTCCTCTTAGGGGCCACT

m*Col1a1*-R: CCACGTCTCACCATTGGGG

m*Col2a1*-F: GGGTCACAGAGGTTACCCAG

m*Col2a1*-R: ACCAGGGGAACCACTCTCAC

m*Dnm3os*-F: CAAGGCTCTCACTTGTCCTG

m*Dnm3os*-R: CAGCTGGAAACTGACCAAAG

m*Lfi35*-F: GTGACCCTGCAAACTGTCCTC

m*Lfi35*-R: TTCAGGTACTGAGAATGGGATCT

m*Lfi47*-F: TCTCCAGAAACCCTCACTGGT

m*Lfi47*-R: TCAGCGGATTCATCTGCTTCG

m*Lfit1*-F: CTGAGATGTCACTTCACATGGAA

m*Lfit1*-R: GTGCATCCCCAATGGGTTCT

m*Lifh1*-F: AGATCAACACCTGTGGTAACACC

m*Lifh1*-R: CTCTAGGGCCTCCACGAACA

m*Stat1*-F: TCACAGTGGTTCGAGCTTCAG

m*Stat1*-R: GCAAACGAGACATCATAGGCA

m*Sfrp2*-F: CGTGGGCTCTTCCTCTTCG

m*Sfrp2*-R: ATGTTCTGGTACTCGATGCCG

m*Rsad2*-F: GCAGAGATGGACGATATGAGAGG

m*Rsad2*-R: GCTGAGTGCTGTTCCCATCT

m*Nmi*-F: GCAGAGATGGACGATATGAGAGG

m*Nmi*-R: CGACTGCAATTCAGCTTCAAGTT

m*Ngf*-F: TGATCGGCGTACAGGCAGA

m*Ngf*-R: GCTGAAGTTTAGTCCAGTGGG

m*Mmp3*-F: ACATGGAGACTTTGTCCCTTTTG

m*Mmp3*-R: TTGGCTGAGTGGTAGAGTCCC

m*Lif*-F: GCCCCAGAAGTAAAACCTTCAG

m*Lif*-R: CCTTCCATTTCTCTCCATTCCAA

m*Flt4*-F: CTGGCAAATGGTTACTCCATGA

m*Flt4*-R: ACAACCCGTGTGTCTTCACTG

m*Cxcl10*-F: CCAAGTGCTGCCGTCATTTTC

m*Cxcl10*-R: GGCTCGCAGGGATGATTTCAA

m*Mmp9*-F: CTGGACAGCCAGACACTAAAG

m*Mmp9*-R: CTCGCGGCAAGTCTTCAGAG

### Immunofluorescence staining and RNA-FISH

Cultured cells were fixed in 4% paraformaldehydein PBS for 15 min at room temperature, and blocked for 30 min in PBS, 3%BSA (Biofroxx,4240), and 0.3% Triton X-100 (Biosharp, BS084) prior to overnight incubation with primary antibodies. Anti-Sox9 (Cell Signaling Technology, D8G8H), anti-Col2a1 (Bioss Antibodies, bs-10589R), anti-Mmp13 (Bioss Antibodies, bs-10581R) and Collagen10a1 (Biorbyt, orb221376) were used at 1:1000. Cells grown on cover slips for RNA-FISH were fixed in 3.7% formaldehyde solution at room temperature for 10 minutes, then permeabilized with 70% cold ethanol for at least an hour. Stellaris probe was added in 100 µL of hybridization buffer (Biosearch, Inc.) and incubated in a dark humidified chamber at 37 °C for 4 h. The cells were visualized with DAPI (Sigma,MBD0015) counterstaining (5 ng/mL) under a wide field fluorescence microscope.

### Edu incorporation assay

To measure cell growth, 24 hours after transfection in 24-well plates, 20 mM EdU (Ribobio,C10310-1) was added for 8 hour. The cells were then fixed in 3.7% formaldehyde, then washed with PBS and permeablized. 500 µl Click-iT reaction cocktail (430 ml 1xClick-iT reaction buffer, 20 ml CuSO_4_, 1.2 ml Alexa Fluor® azide, and 50 ml reaction buffer additive) was added to each well and incubated for 45 min at the room temperature in the dark. The cells were counter-stained with DAPI for nuclei and visualized under an inverted fluorescence microscope. Images were processed with Image J and the percentage of EdU incorporation was calculated based on the number of EdU positive (red) and total (DAPI) cells.

### Alcian blue staining and quantification

Chondrocytes were cultured for 14 days in chondrogenic differentiation medium, then fixed in 4% formalin for 10 min. After washing twice with PBS, the cells were incubated with 3% acetic acid for 10 min and stained with 1% alcian blue in 3% acetic acid (pH 2.5) for 30 min and photographed. For quantification, the stained cells were washed twice, and the alcian blue dye was extracted with 500 ml dimethyl sulfoxide (Sigma,D8418). Absorbance was measured at 650 nm.

## Alkaline phosphatase assay

Histochemical detection of alkaline phosphatase activity was performed on cells that were fixed for 2 min in 4% paraformaldehyde at room temperature. After washing with TBST, the cells were incubated for 30 min in in 0.1 M Tris-HCl, pH 8.5, containing 0.1 mg/ml Naphthol AS-MX phosphate (Sigma,N4875), 0.5% N,N-dimethylformamide (Sigma,D4551), 2 mM MgCl_2_, and 0.6 mg/ml fast blue BB salt (Sigma,D9805), and then photographed.

### In vitro **differentiation of chondrogenic ATDC5 cells**

ATDC5 cell line was culture in DMEM/F-12 medium supplemented with 5% FBS, penicillin (100units/mL)/streptomycin (0.1 mg/mL), and 4mM L-Glutamine (Gibco,25030-081). For differentiation experiments, ATDC5 cells were seeded at 80–90% confluence in 12-well plate. After reaching 100% confluence, ATDC5 cells were incubated with serum-free medium for 24 h, then exposed to differentiating medium containing 1% Insulin-transferrin-sodium selenite (ITS, Sigma-Aldrich) and 50nM Vitamin C (Sigma,A4544).

### RNA sequencing and data analysis

RNA from Rk5-vector, Full length and DKO mutant *Dnm3os* transfected primary mouse articular chondrocytes were extracted using TRIzol (Life Technol- ogies), followed by purification using a RNeasy Mini Kit (Qiagen,74104). RNA-seq was performed using primary mouse articular chondrocytes from two individual animals. RNA-seq libraries were prepared using the Illumina TruSeq RNA Library Prep Kit v2 and sequenced by a HiSeq 2500 sequencer. RNA-seq reads were aligned to mm10 using TopHat with default settings (.cbcb.umd.edu/).

### Statistical analysis

Prism 8.0 (GraphPad software) was employed for analyses. Data from a minimum of three independent experiments were presented as mean ± s.d. Animals in the experiment were randomly selected and grouped. An unpaired two-tailed Student’s t-test was used for analyzing two data sets, and one-way analysis of variance was used for more sets. The significance threshold was set at 0.05 (*p* < 0.05).

## Supplementary Information

**Additional file 1.** Clinical description of the proband.

**Additional file 2: Fig. S1.** Body weight, body length and morphometric characteristics skull in miR-214 KO mice. Body weight (A) and body length (B) of miR-214 KO mice (n=15) and their WT littermates (n = 18) measured starting from 6 week to 24 weeks of age. Body weight and body length of the KO mice were modestly smaller than the WT mice at most time-points examined, the difference was not statistically significant. Morphometric characteristics skull width(C), skull length (D), skull width/length, and inner canthal distance (E) of WT and miR-214 KO, the difference was not statistically significant.

**Additional file 3: Fig. S2.*** miR-214* KO drastically increase the height relative genes. qPCR expression analysis of Noonan syndrome relative genes in miR-214 KO blood , miR-214 Het relative to WT mice (n=5 in each genotype). Data shown are the fold induction of gene expression normalized with Hprt and expressed as mean ± S.E.M. One-way ANOVA test was used for statistical analysis. ** P<0.01, *** P<0.001, and ns, not significant

**Additional file 4: Table S1.** Predicted miR-214 and miR-199a recognition sites.

**Additional file 5: Fig. S3.** LncRNA-Dnm3os impedes chondrocyte differentiation while promotes the proliferation. RT-qPCR quantification of* Sox9* in primary articular chondrocytes at passages as noted. (B) RT-qPCR quantification and statistical analysis of* Col1a1* and* Col2a1* in primary articular chondrocytes transfected with sh*Dnm3os* at passages as noted. (C) EdU labeling of proliferating chondrocytes transfected with scrambled shRNA, sh*Dnm3os* and sh*Dnm3os* together with DKO. (D) RT-qPCR quantification of Dnm3os in (C). (E) statistical analysis of EdU positive cells in (C). (F) RT-qPCR quantification of* Col10a1* and (G)* Mmp13* in primary articular chondrocytes transfected with scrambled shRNA, shDnm3os and shDnm3os together with DKO after 2 weeks chondrocyte differentiation. (H-I) RT-qPCR quantification of overexpression level of Dnm3os in primary articular chondrocytes and ATDC5 cells transfected with RK5, FL and DKO. *, P<0.05, **, P<0.01, ***, P<0.001.

**Additional file 6: Fig. S4.** Forced expression of* miR-214* and* miR-199a* impedes chondrocyte differentiation while promotes the proliferation. Confocal images of GFP and IF staining of* Col2a1* in primary articular chondrocytes after the cells were transfected with P2GM vector, P2GM-miR-199a (P199), or P2GM-miR214 (P214) and differentiated for 2 weeks. (B) RT-qPCR quantification of Col2a1 or (C)* Col10a1* in cells of (A). (D) the same as in (A) except for the staining of Sox9 and the cells were quantified for* Sox9* (E) and (F)* Mmp13* by RT-qPCR. (G) and (H) EdU staining of cells as in (A) and quantification thereof. Student T-test was used for statistical analysis. *, P<0.05, **, P<0.01, ***, P<0.001.

**Additional file 7: Fig. S5.** Forced expression of* Dnm3os* or* Dnm3os*-DKO downregulates height-related genes in mouse primary articular chondrocytes. RT-qPCR analysis of Noonan syndrome genes in primary chondrocytes that were transfected with pRK5-*Dnm3os* or pRK5-*Dnm3os*DKO. Data shown are the fold induction normalized against Hprt and are expressed as mean ± S.E.M. One-way ANOVA test was used for the statistical analysis. ** P<0.01, *** P<0.001, and ns, not significant.

**Additional file 8: Fig. S6.** Insulin-supplemented differentiation medium cultivation induces differentiation of ATDC5 cells. (A) Alcian blue staining of cartilage matrix produced by differentiated ATDC5 cells after 18-days differentiation. (B-C) RT-qPCR quantifications of Col1a1, Col2a1 in ATDC5 cells at different days of differentiation as noted. (D) RT-qPCR quantifications of Dnm3os in ATDC5 cells at different days of differentiation as noted. (E) RT-qPCR quantifications of Sox9 in ATDC5 and iMAC cells transfected with RK5, FL and DKO. (G) Top 20 enriched pathways in FL and DKO transfected primary articular chondrocytes. The size and color of the dots represent the enriched gene number and the range of -log10(p values), respectively. Student T-test was used for statistical analysis. *, P<0.05, **, P<0.01, ***, P<0.001.

**Additional file 9: Table S2.** shRNA sequence of Dnm3os.

## Data Availability

All data generated or analysed during this study are included in this published article and its additional files.

## Abbreviations

MAPK: Mitogen-activated protein kinase
ncRNA: Noncoding RNA
lncRNAs: Long non-coding RNAs
NGF: Nerve growth factor
DNM3: Dynamin 3
Dnm3os: DNM3 opposite strand
TMJ: Temporomandibular joint
MSCs: Mesenchymal stem cells
