# Supplementary material for: Deletion at an 1q24 locus reveals a critical role of long noncoding RNA DNM3OS in skeletal development
Source: Cell Biosci. 2021 Mar 2;11:47. doi: 10.1186/s13578-021-00559-8 (PMC7923828; doi:10.1186/s13578-021-00559-8)
Supplement: Supplementary file 1 — Additional file 1. Clinical description of the proband. [file 13578_2021_559_MOESM1_ESM.docx]

**Clinical description of the proband**

The proband had been born to a 21-year-old G3P2 mother following a 39-week pregnancy. There was exposure to about half a pack of cigarettes per day during the pregnancy. Birth was by normal spontaneous vaginal delivery. Birth weight was 6 pounds (between the 5^th^ and 10^th^ percentiles). Birth length was 18 inches (3^rd^ percentile). He required home phototherapy for jaundice but was otherwise healthy as a newborn.

By six months, his length and weight were below the fifth percentile. By twelve months, they had fallen below the first percentile. At 16 months, he had normal proportions by lower segment and arm length measurements. At 18 months he presented to Genetics clinic for evaluation (Fig). Length was 3.4 SD below the mean for age. Middle finger-to-hand length ratio of 0.39 was between the 3^rd^ and 25^th^ percentiles. He had fleshy palms with single transverse palmar creases. He had a high columella, downslanting palpebral fissures, and downturned corners of the mouth. He was relatively macrocephalic and had a protuberant but soft belly without hepatosplenomegaly. We noted several poorly defined hyperpigmented macules over the trunk and extremities. His review of systems revealed a history of multiple episodes of otitis media necessitating PE tube placement and mildly excessive bleeding after tonsillectomy.

By four years two months his height was 3.7 SD below the mean for age. Middle finger-to-hand length ratio had fallen to 0.37 (below the 3^rd^ percentile). Upper-to-lower segment ratio was increased at 1.16, and arm span of 84.5 cm was shorter than expected for height of 87.7 cm. Head circumference was at the 57^th^ percentile.

At 4 ½ years of age growth hormone stimulation testing showed evidence of hypothalamic dysfunction. An IGF-1 generation study was also positive, and he has been started on growth hormone.

He had normal development and behavior until the age of 3 ½, when a school evaluation showed mild fine motor delay. He had minor oppositional behaviors at 4 years 2 months but has not been formally evaluated from a behavioral standpoint.

Radiographic investigations in the proband included two normal skeletal surveys at 16 months and 3 ½ years; delayed bone age at two years with normalization of the bone age by 3 ½ years; normal MRI of the brain; normal echocardiogram; and normal kidney ultrasound. Additional laboratory investigations included normal parathyroid hormone, calcium, TSH, and thyroxine levels; normal sweat chloride testing; mildly elevated urine mucopolysaccharides with normal qualitative analysis; normal dilated eye exam; normal screening for celiac disease; normal MMC- and DEB-related chromosomal breakage; normal Russell Silver testing (chromosome 7 disomy analysis and DMR1 methylation studies); normal sequencing and deletion/duplication analysis of *SHOX*; normal 46,XY G-banding study; and normal oligonucleotide-based testing for variants in *BRAF*, *HRAS*, *KRAS*, *MEK1*, *MEK2*, *NRAS*, *PTPN11*, *RAF1*, *SOS1*, *CBL*, and *SPRED1* and for the recurrent Noonan-related variant in *SHOC2*.

**Family History**

The proband’s mother is 5’2” and has a distinctive appearance including broad nasal base, short nose, pointed chin, full lips, and generous facial freckling (Fig). His father is 6’2” with no unusual features.

The proband is the second of two children born to his biological parents together. His older full brother has normal stature and development. There is one maternal half-sister, and five paternal half-siblings, all of whom are reported to have normal stature and development for age.

The maternal grandfather is 5’7”. A maternal half-uncle has a history of ADHD. There are no other individuals reported with short stature or cognitive impairment. The family history as reported is negative for birth defects, hearing or vision impairment. Parental consanguinity is denied.
