# Supplementary figures and images for: Deletion at an 1q24 locus reveals a critical role of long noncoding RNA DNM3OS in skeletal development
Source: Cell Biosci. 2021 Mar 2;11:47. doi: 10.1186/s13578-021-00559-8 (PMC7923828; doi:10.1186/s13578-021-00559-8)

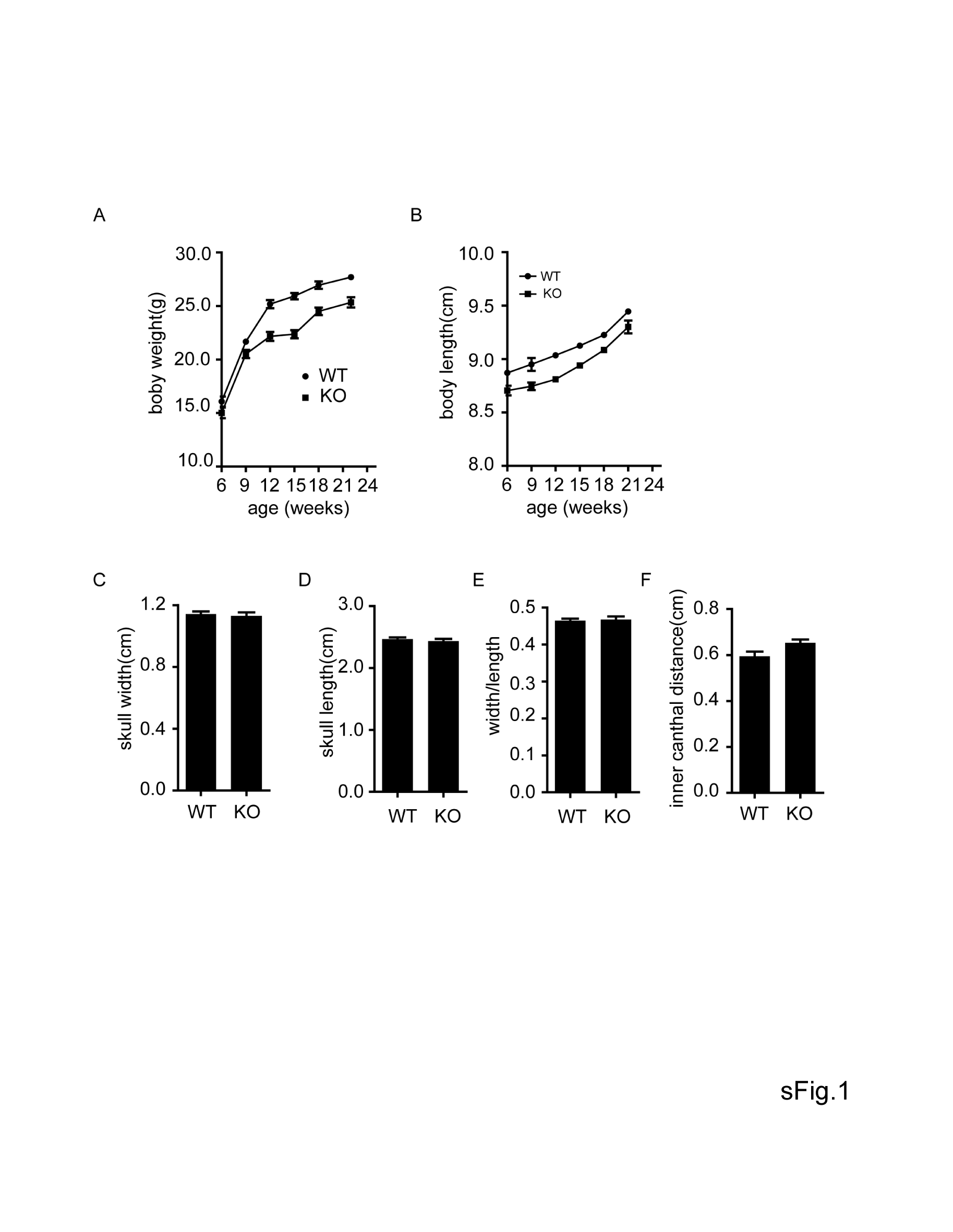

Supplement: Supplementary file 2 — Additional file 2: Fig. S1. Body weight, body length and morphometric characteristics skull in miR-214 KO mice. Body weight (A) and body length (B) of miR-214 KO mice (n=15) and their WT littermates (n = 18) measured starting from 6 week to 24 weeks of age. Body weight and body length of the KO mice were modestly smaller than the WT mice at most time-points examined, the difference was not statistically significant. Morphometric characteristics skull width(C), skull length (D), skull width/length, and inner canthal distance (E) of WT and miR-214 KO, the difference was not statistically significant. [file 13578_2021_559_MOESM2_ESM.tif]

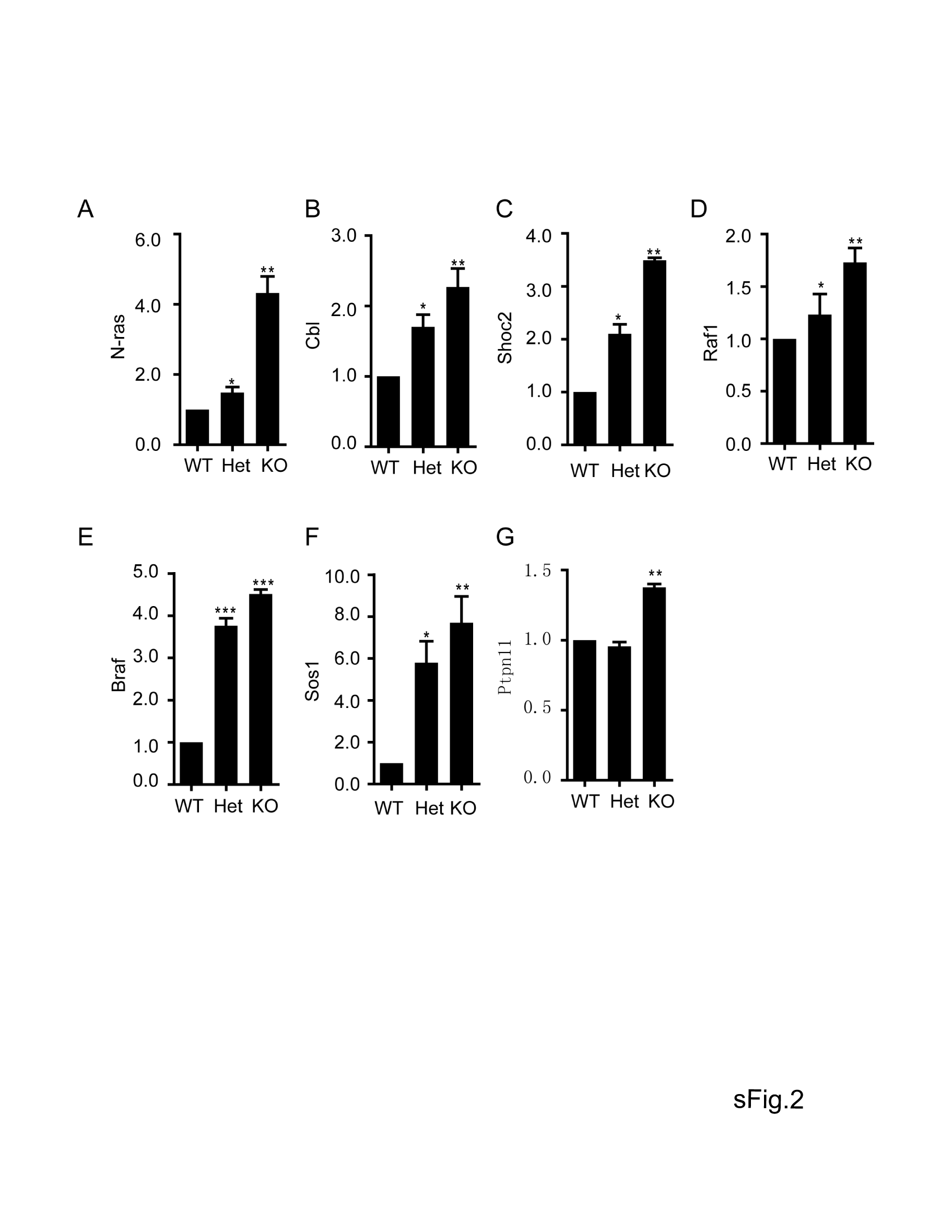

Supplement: Supplementary file 3 — Additional file 3: Fig. S2. miR-214 KO drastically increase the height relative genes. qPCR expression analysis of Noonan syndrome relative genes in miR-214 KO blood , miR-214 Het relative to WT mice (n=5 in each genotype). Data shown are the fold induction of gene expression normalized with Hprt and expressed as mean ± S.E.M. One-way ANOVA test was used for statistical analysis. ** P<0.01, *** P<0.001, and ns, not significant [file 13578_2021_559_MOESM3_ESM.tif]

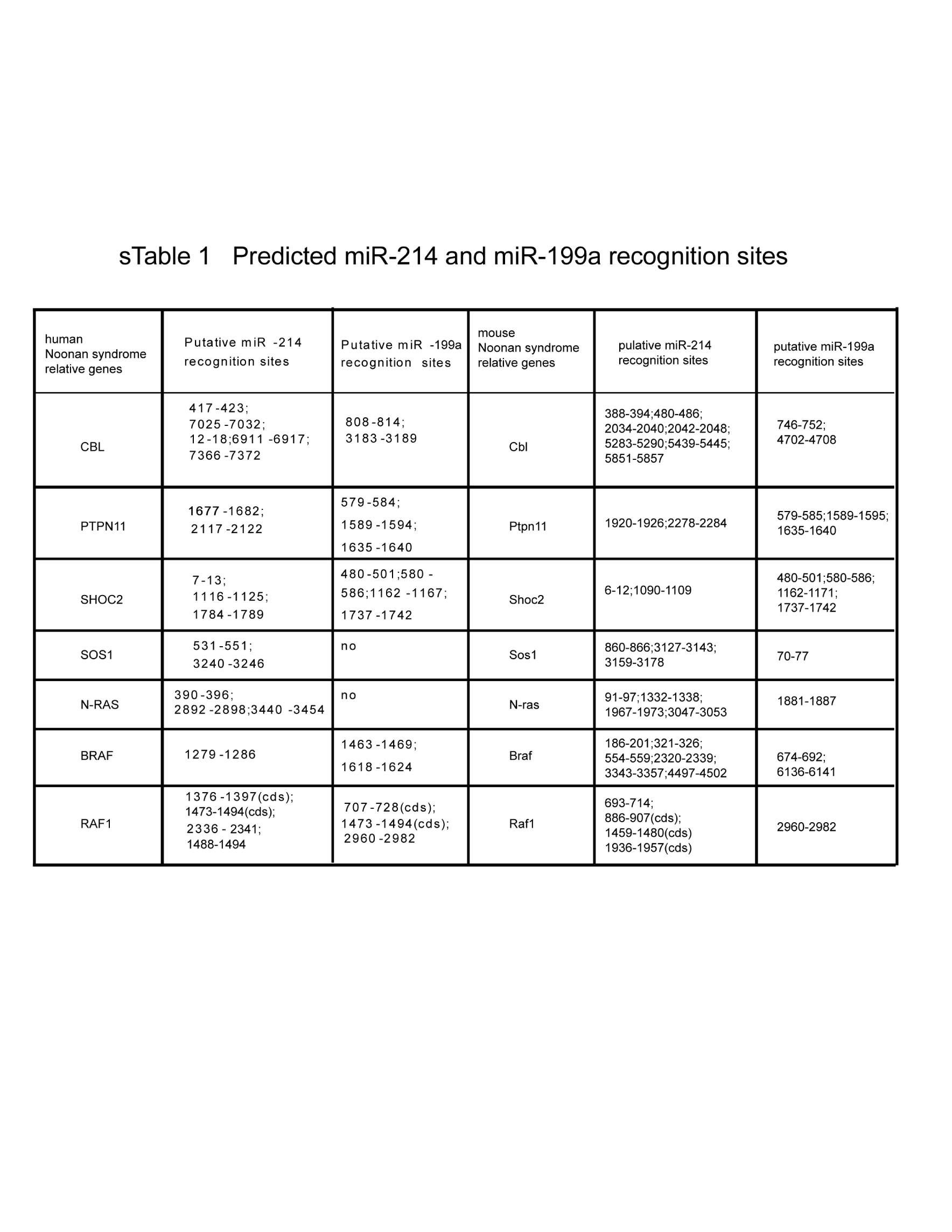

Supplement: Supplementary file 4 — Additional file 4: Table S1. Predicted miR-214 and miR-199a recognition sites. [file 13578_2021_559_MOESM4_ESM.tif]

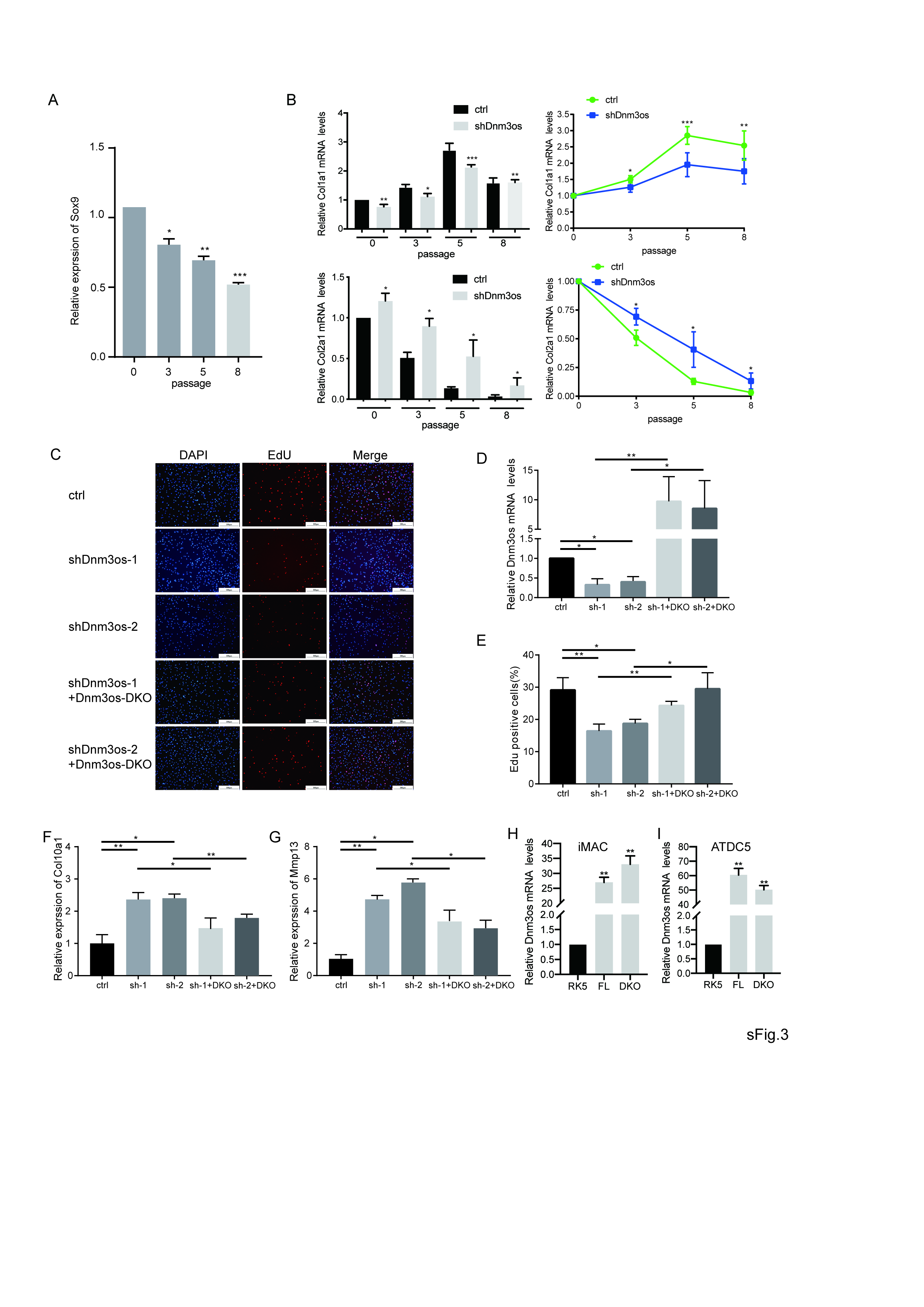

Supplement: Supplementary file 5 — Additional file 5: Fig. S3. LncRNA-Dnm3os impedes chondrocyte differentiation while promotes the proliferation. RT-qPCR quantification of Sox9 in primary articular chondrocytes at passages as noted. (B) RT-qPCR quantification and statistical analysis of Col1a1 and Col2a1 in primary articular chondrocytes transfected with shDnm3os at passages as noted. (C) EdU labeling of proliferating chondrocytes transfected with scrambled shRNA, shDnm3os and shDnm3os together with DKO. (D) RT-qPCR quantification of Dnm3os in (C). (E) statistical analysis of EdU positive cells in (C). (F) RT-qPCR quantification of Col10a1 and (G) Mmp13 in primary articular chondrocytes transfected with scrambled shRNA, shDnm3os and shDnm3os together with DKO after 2 weeks chondrocyte differentiation. (H-I) RT-qPCR quantification of overexpression level of Dnm3os in primary articular chondrocytes and ATDC5 cells transfected with RK5, FL and DKO. *, P<0.05, **, P<0.01, ***, P<0.001. [file 13578_2021_559_MOESM5_ESM.tif]

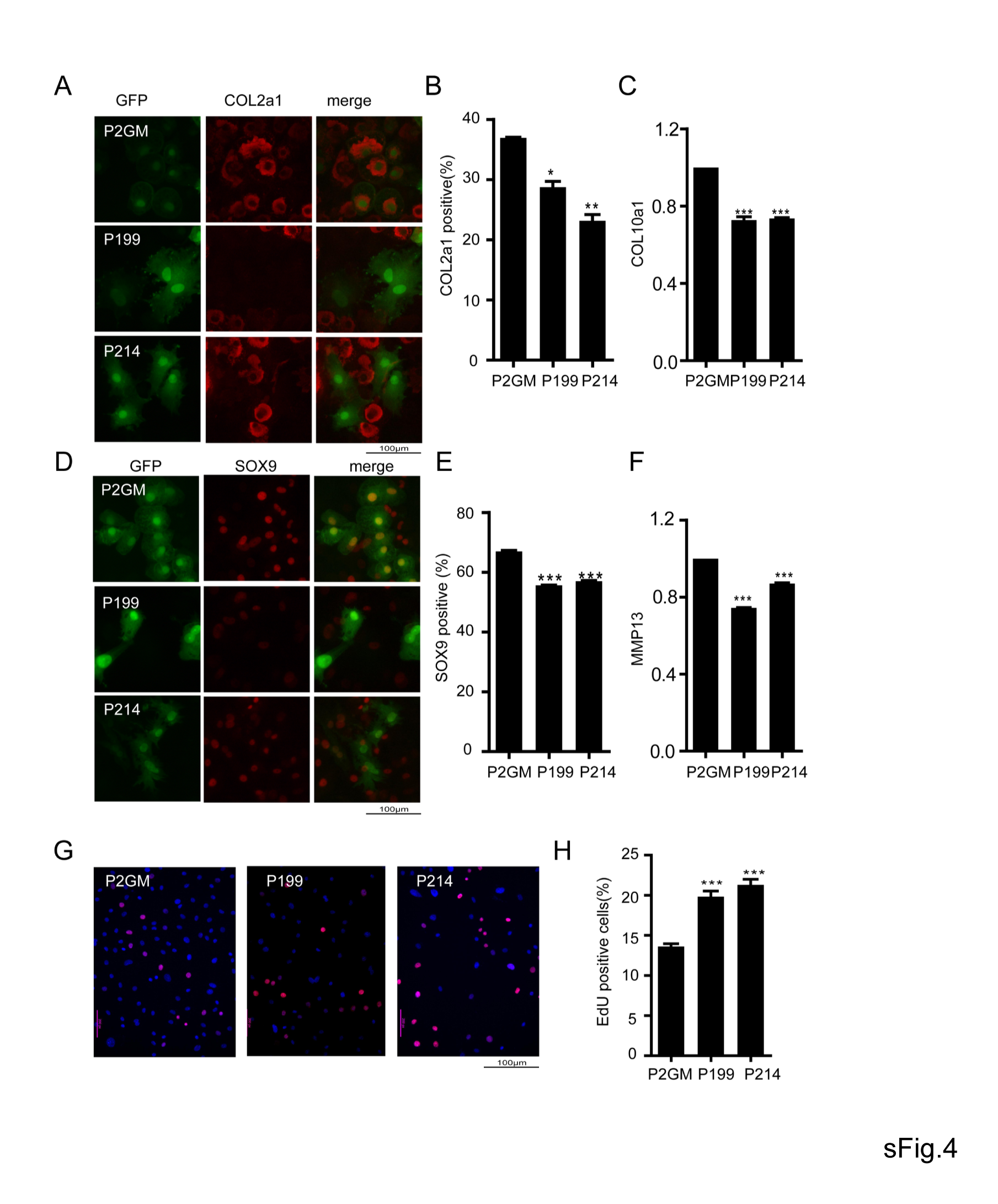

Supplement: Supplementary file 6 — Additional file 6: Fig. S4. Forced expression of miR-214 and miR-199a impedes chondrocyte differentiation while promotes the proliferation. Confocal images of GFP and IF staining of Col2a1 in primary articular chondrocytes after the cells were transfected with P2GM vector, P2GM-miR-199a (P199), or P2GM-miR214 (P214) and differentiated for 2 weeks. (B) RT-qPCR quantification of Col2a1 or (C) Col10a1 in cells of (A). (D) the same as in (A) except for the staining of Sox9 and the cells were quantified for Sox9 (E) and (F) Mmp13 by RT-qPCR. (G) and (H) EdU staining of cells as in (A) and quantification thereof. Student T-test was used for statistical analysis. *, P<0.05, **, P<0.01, ***, P<0.001. [file 13578_2021_559_MOESM6_ESM.tif]

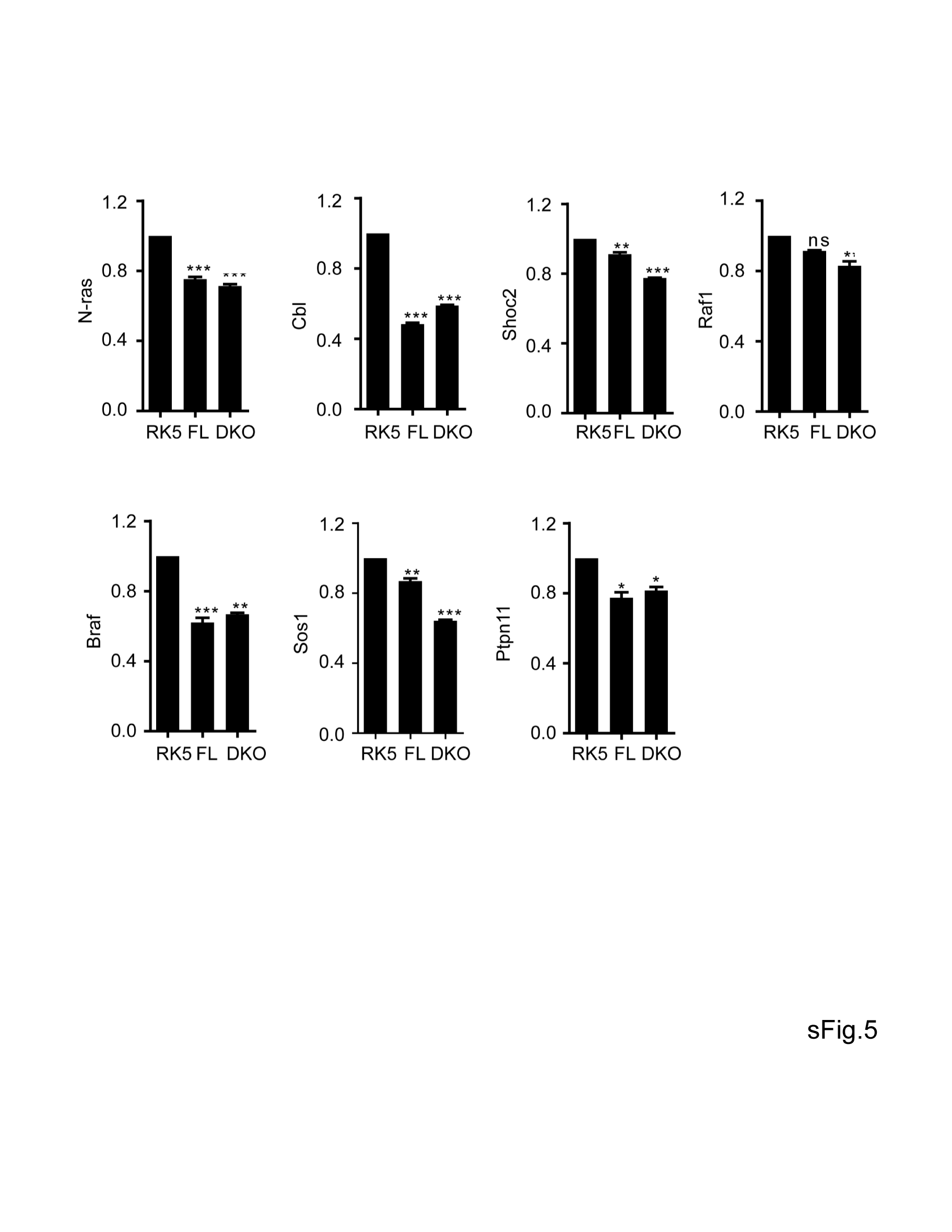

Supplement: Supplementary file 7 — Additional file 7: Fig. S5. Forced expression of Dnm3os or Dnm3os-DKO downregulates height-related genes in mouse primary articular chondrocytes. RT-qPCR analysis of Noonan syndrome genes in primary chondrocytes that were transfected with pRK5-Dnm3os or pRK5-Dnm3osDKO. Data shown are the fold induction normalized against Hprt and are expressed as mean ± S.E.M. One-way ANOVA test was used for the statistical analysis. ** P<0.01, *** P<0.001, and ns, not significant. [file 13578_2021_559_MOESM7_ESM.tif]

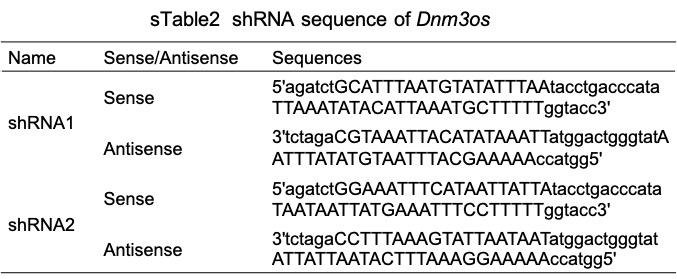

Supplement: Supplementary file 9 — Additional file 9: Table S2. shRNA sequence of Dnm3os. [file 13578_2021_559_MOESM9_ESM.tif]
